# 

**Published:** 1900-04

**Authors:** 


### CONTENTS:

### SELECTIONS.

Article I.—An Evening with Bonwill. I. Norman Broomell, D. D. S .......... 529

" II.—Report of the Pennsylvania Association of Dental Surgeons. Theodore F. Chupein .......... 537

" III.—Nitrous Oxid With Air or With Oxygen .......... 539

" IV.—Facial Deformities. W. E. Davis, D. D. S .......... 541

" V.—The Details of Constructing a Porcelain Bridge. G. W. Schwartz, M. D., D. D. S .......... 549

" VI.—Disease of the Maxillary Antrum Successfully Treated Through a Root Canal. Cuvier R. Marshall, A. M., M. D .......... 556

" VII.—Arsenic in Oxyphosphates Not Injurious. Dr. W. V. B. Ames .......... 559

" VIII.—Oxyphosphate Fillings not Injurious. Prof. G. S. Junkerman .......... 564

" IX.—Examining Boards Should be Controlled by State Societies .......... 567

### MONTHLY SUMMARY.

Microbes in the Arctic Regions .......... 569

The Sterilization of Water by Means of Ozone .......... 570

A New Alloy .......... 570

Plain Teeth With Pink Rubber .......... 571

The Useful Lemon .......... 571

Sensitive Dentine .......... 572

Dentistry in France .......... 572

Chloroform in India .......... 572

"Dentistry" vs "Mechanical Dentistry" .......... 573

A Young Licentiate of Quebec .......... 573

Skulls and Brain Capacity .......... 574

Repairing Rubber Plates .......... 574

Real Diamonds .......... 574

Sterilizing Dental Instruments .......... 575

Anchylosis of the Jaw Due to Interstitial Myositis .......... 575

Legrand's Solution Anesthesique Hemostatique .......... 576

